# Frontline health workers and exclusive breastfeeding guidelines in an HIV endemic South African community: a qualitative exploration of policy translation

**DOI:** 10.1186/s13006-018-0164-y

**Published:** 2018-06-07

**Authors:** Sara Nieuwoudt, Lenore Manderson

**Affiliations:** 1 School of Public Health, University of the Witwatersrand, 1 Jan Smuts Avenue, Braamfontein, Johannesburg, 2000 South Africa; 2 Developmental Pathways Health and Research Unit, University of the Witwatersrand, 1 Jan Smuts Avenue, Braamfontein, Johannesburg, 2000 South Africa; 3 Institute at Brown for Environment and Society, Brown University, 85 Waterman St, Providence, RI 02912 USA

**Keywords:** Exclusive breastfeeding, Breastfeeding promotion, Health workers, Policy analysis, Qualitative research, South Africa

## Abstract

**Background:**

Mothers rely heavily on health worker advice to make infant feeding decisions. Confusing or misleading advice can lead to suboptimal feeding practices. From 2001, HIV positive mothers in South Africa were counseled to choose either exclusive breastfeeding or exclusive formula feeding to minimize vertical HIV transmission. On the basis of revised World Health Organization guidelines, the government amended this policy in 2011, by promoting exclusive breastfeeding and discontinuing the provision of free formula. We explored how health workers experienced this new policy in an HIV endemic community in 2015–16, with attention to their knowledge of the policy, counselling practices, and observations of any changes.

**Methods:**

We interviewed eleven health workers, from four community health clinics, who had counseled mothers before and after the policy change. The transcribed interviews were analyzed thematically, using a hybrid coding approach.

**Results:**

The scientific rationale of the policy was not explained to most health workers, who mostly thought that the discontinuation of the formula program was cost-related. The content of their counseling reflected knowledge about promoting breastfeeding for all women, and accordingly they mentioned the nutritional and developmental benefits of breastfeeding. The importance of exclusive breastfeeding for all infants was not emphasized, instead counseling focused on HIV prevention, even for uninfected mothers. The health workers noted an increased incidence of breastfeeding, but some worried that to avoid HIV disclosure, HIV positive mothers were mixed feeding rather than exclusively breastfeeding.

**Conclusions:**

Causal links between the policy, counseling content and feeding practices were unclear. Some participants believed that breastfeeding practices were driven by finance or family pressures rather than the health information they provided. Health workers generally lacked training on the policy’s evidence base, particularly the health benefits of exclusive breastfeeding for non-exposed infants. They wanted clarity on their counseling role, based on individual risk or to promote exclusive breastfeeding as a single option. If the latter, they needed training on how to assist mothers with community-based barriers. Infant feeding messages from health workers are likely to remain confusing until their uncertainties are addressed. Their insights should inform future guideline development as key actors.

## Background

All children deserve a chance not just to survive, but to thrive. The way that they are fed in the first 1000 days, particularly the first six months, plays a part in setting them on particular health and development trajectories [1]. The World Health Organization (WHO) has endorsed exclusive breastfeeding (EBF) for the first six months as optimal for all infants [2–4], yet in 2016 only an estimated 37% of infants in low and middle income countries were exclusively breastfed [5]. The definition of EBF is feeding only breastmilk, with no other liquids or solids, save prescribed medications or vitamins [6].

In the complex social ecology of how infant feeding decisions are made [7], health workers (HWs) play a central role. In studies of decision making on infant care in South Africa, mothers have consistently referred to the importance of the advice they receive from HWs [8, 9]. Health worker advice, when consistent, can assist mothers to persevere against community norms or family pressure to mixed feed [10, 11]. Likewise, inconsistent or misguided feeding advice can confuse mothers and contribute to suboptimal practices [8, 9]. The employment of different cadres of HWs to counsel mothers from clinic sisters to community health workers [12], has increased the need to focus on the consistency and quality of infant feeding counseling reaching mothers as they interact with the health system from the ante to postnatal period [13, 14].

**Fig. 1:**
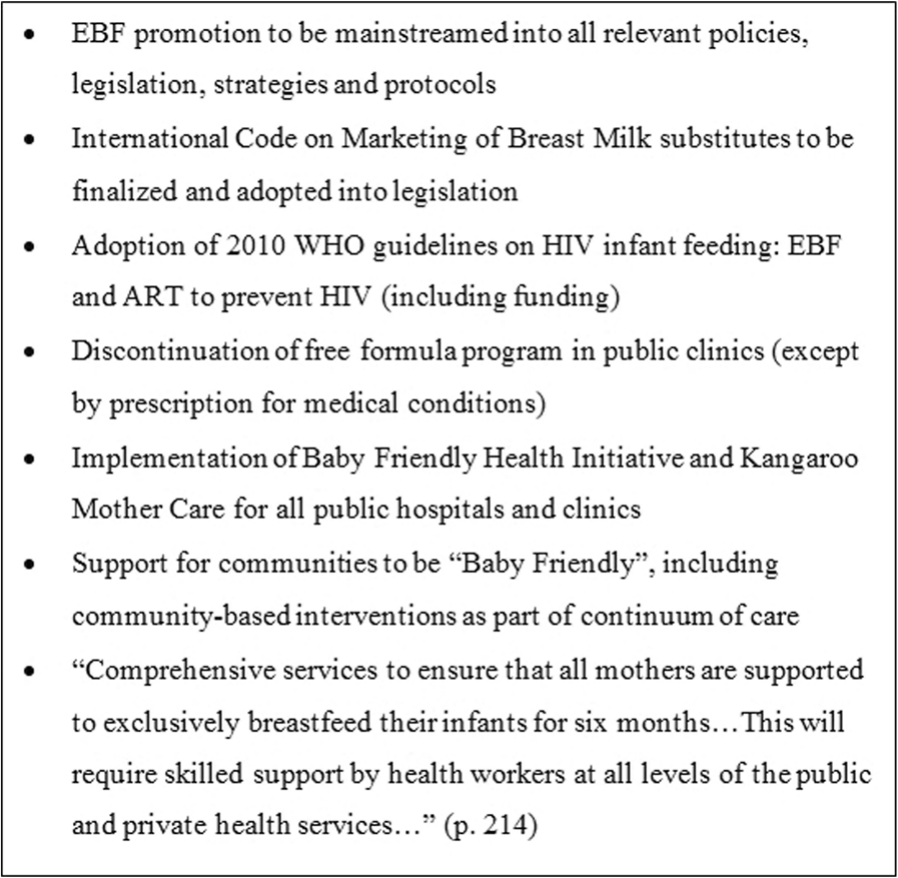
Tshwane Declaration Highlights

A policy feature of South Africa is that exclusive formula feeding (EFF) for six months, also known as replacement feeding, was supported as part of the prevention of mother to child transmission (PMTCT) program from 2001 to 2011; EFF was deemed optimal for HIV exposed infants during that 10-year period [15]. In 2011, however, the Tshwane Declaration of Support for Breastfeeding (hereafter, the Declaration) shifted the infant feeding guidelines, requiring changes to the health system and counseling. The Ministry of Health’s announcement of the Declaration called for unified messaging to support exclusive breastfeeding for all women and to phase out free formula to HIV positive mothers [16] (see Figure. 1). The Declaration followed the WHO’s new guidelines on HIV and infant feeding recommending that countries adopt either EBF with antiretrovirals (ARVs) or EFF [3, 17]. The formula phase out in South Africa was completed in 2012.

The behavioral impact of the Declaration appears to have been significant. By 2016, 31.7% of children under six months were exclusively breastfed [18], compared to less than 10% in the decades preceding the policy change [15]. This occurred despite the challenges associated with changes as a result of the Declaration, in the meaning of what was considered to be consistent counseling for HWs and optimal feeding for HIV positive mothers. The HWs who counseled before and after the change would have been subject to different infant feeding metrics, and the previously “good” practice of EFF for six months for HIV-exposed infants was replaced by an emphasis on EBF for all infants.

While the Declaration heralded higher EBF rates in South Africa, guidelines alone do not produce behavior change [19]. Guidelines need to be translated into a format that can be interpreted and used by HWs [20]. Even then their advice is only one factor as feeding is negotiated within a complex social ecology [1, 7, 19]. Our interest was in how HWs at the primary facility level, positioned at the crossroads of guidelines and communities, experienced this policy change and by implication either inhibited or supported policy translation.

While much is known about the role of HWs prior to the Declaration, less is known about how they responded to the policy change. For two decades HWs were trained in conflicting messages, that breast was “best” for child survival and that HIV could be transmitted from mother to child via breastmilk [21, 22]. During this period, there was clear documentation that HW counseling was not meeting mothers’ needs [23] and mothers were often confused with mixed messages [8, 24, 25]. Many researchers advocated promoting a single feeding option [21], although some feared that this might unintentionally increase mixed feeding among HIV positive mothers [19, 26].

We applied qualitative methods to explore in depth how HWs attached to community health clinics (CHCs) in Soweto, Johannesburg, understood and were implementing the new infant feeding guidelines in late 2015 and early 2016. In this article, we explore 1) HW knowledge of the Declaration; 2) how formula removal and training influenced their counseling; and, 3) their impressions about changes in breastfeeding practices. Drawing on a phenomenological approach and its focus on lived experience [27], we asked HWs to share and reflect upon their upbringing, experiences of infant feeding, and values as these related to their experiences.

This study contributes to policy analysis, particularly policy translation. It is important for policy-makers and public health practitioners to understand how HWs experience and interpret infant feeding guidelines, as they play a critical role overall in infant feeding decision making. This case is particularly interesting because community norms about the intersection of HIV and infant feeding have been formed over more than a decade. The inclusion of HWs who are HIV positive brings to light the power of the personal as it relates to policy.

## Methods

### Study design

This study was embedded within a cross-sectional mixed-methods study called “Infant feeding in the Context of HIV: Soweto, South Africa,” which applied a socioecological framework to explore infant feeding influences in one sub-district of Soweto in the context of HIV. The larger study included a quantitative cross-sectional survey of 298 HIV-infected and uninfected mothers with infants below six months of age who accessed one of the five CHCs in the study sub-district in Soweto. The survey explored infant feeding patterns and associated factors. From this sample, 46 mothers participated in in-depth interviews to explore their decision making processes in greater depth. Three research experts in infant feeding research in South Africa participated in key informant interviews to provide insight on policy guideline shifts. To address the health system level, eleven HWs, three of whom were HIV positive, were recruited from four of the five CHCs, and were interviewed to represent frontline service provider perspective. These interviews are the focus of this paper.

### Study site

Soweto, located in the southwest of Johannesburg, has a population over one million. It was originally reserved for Black South Africans until apartheid ended in 1994, although demographic distribution since then has seen little change. At the time this study commenced, Johannesburg had antenatal HIV rates of nearly 30% [28]. Health workers were recruited from four of the five busy provincial CHCs serving the study sub-district, with the aim of interviewing three to five HWs from each facility. The manager of the fifth CHC in the sub-district was also approached for permission to interview health workers, but she was unwilling to provide permission given the negative impact of previous (unrelated) studies on staff morale.

### Sample

The study population was frontline HWs who influenced infant feeding decisions. Inclusion was open to any clinic staff member directly involved in counseling mothers on infant feeding; this included health center employees and non-governmental staff formally seconded to the clinics. The Primary Investigator (PI, first author) and two research assistants (RAs) purposively recruited eligible HWs, based on the facility manager recommendations. To be eligible, study participants had to be involved in counseling both before and after the Declaration, as we wanted to explore their experiences of the introduction of the guidelines and the influence of this on their interactions with women. Three distinct cadres of HWs were identified:

- Professional nurses (2): Stationed in antenatal, postnatal and labor wards, they conduct group health talks and individual counseling about infant feeding and demonstrate feeding techniques. In the labor ward, they ensure mothers can feed their infants either breastmilk or formula prior to discharge; this includes breastfeeding support by ensuring latching, demand feeding and managing milk let down and supply. According to one nurse (CHC4), this was usually accomplished within six hours for breastfeeding mothers; women who formula fed took longer to discharge, as infants also had to pass meconium. Mothers who had a cesarean section also took longer to discharge because of recovery.
- Ward-based Outreach Teams (5): WBOTs are linked to the clinic and managed by government, with responsibility for home-based visits in the surrounding community [29, 30]. They observe infant feeding and refer complicated cases to nurses, social workers, or Mentor Mothers [31]. We identified no formal role for WBOTs to counsel pregnant mothers to breastfeed in this study. In other health contexts, WBOTs would be recognized as community health workers.
- Seconded NGO employees (4): HIV South Africa (HIVSA) employees support nurses with health education in antenatal and postnatal wards. Mentor Mothers (MMs) are HIV positive women trained by Mothers2Mothers as community health workers to support mothers attending the clinic, both inside and outside the clinic setting [31]. All MMs we interviewed were previously clinic staff.

We conducted 11 semi-structured interviews, using a guide, between October 2015 and March 2016. Based on the richness of the interview content and convergence of findings related to their experiences, we elected to publish results, although sampling targets were unmet due to ethical approvals expiring. As targets were not met, we did not compare clinic environments, where three from each site would have been needed as a minimum.

### Data collection

Most interviews were individual, led by the PI or one of the RAs; the second author joined one interview, with the participant’s consent. Both RAs were residents of Soweto: one had lived there her entire life while the other had moved there as an adult. All interviews were conducted in English and audio-recorded. In two instances, RAs supported the PI because the interviewees preferred to speak in isiZulu or Setswana at certain points. Two interviews took place at the study office, one at a NGO worker’s home and the remainder took place in CHC facilities, either in private rooms or private covered outdoor areas. All eligible HWs who we approached agreed to participate.

The PI took field notes and debriefed with RAs during data collection as part of a grounded approach to incorporate new insights into the data collection process, so that beyond deductive lines of inquiry the team was able to identify iterative themes inductively. Debriefing meetings were recorded. All recordings were transcribed and translated by a professional service and quality checked by the PI (and RAs for local language). English transcripts were then imported into NVivo 10.0.

### Data analysis

The PI applied thematic content analysis, using a hybrid of inductive and deductive coding [32, 33]. An initial framework for the codebook was created based on the study objectives to guide detailed exploration of concepts like policy knowledge and counseling content. The transcripts were then coded inductively using constant comparison, drawing on the grounded approach, to identify participants’ experiences. The PI analyzed and grouped the codes into concepts, discussed these with the second author, and further refined them into the themes and sub-themes presented here. Anonymised data are available on request from the authors.

## Results

The eleven HWs ranged in age from 29 to 62 years. One was an auxiliary nurse and another was an advanced professional midwife. Six had matriculated from high school; the remaining three, all WBOTs, had not finished high school. In addition to being professionals, they also were mothers who at some stage in their life had to grapple with their own infant feeding decisions related to mixed feeding and duration of breastfeeding, three had a known HIV positive status and had directly experienced the changing guidelines pertaining to PMTCT. They drew on these personal experiences to support the women they counseled. As per the eligibility criteria, the HWs had been involved in infant feeding counseling before the guideline went into effect, although some had held different positions to the ones they held at the time of the interviews, such as community health workers. The amount of time they had been involved in counseling infant feeding practices ranged from five to 33 years.

### Policy knowledge: Mostly organic

There was uncertainty about the policy’s origin. Nurses, WBOTs and NGO staff all believed cost was a significant reason for ending the formula program. WBOTs explained that “the government can’t afford it” or “can’t keep giving.” A minority believed that the health system changes were the result of women cheating the program to get extra formula, as one nurse explained:

*It’s a good thing that it stopped because those mothers they were so greedy most of them … they were using the cards to come and collect their milk and then she will come and tell that the card is vanished in the house. “I can’t see it.” They make another card. The person having two cards. Now she’ll come with this card to collect the milk. Next time, the other card.* (Nurse, CHC4).

Most were unclear about why the formula program had ended.

Specific training on the Declaration was reported only by three NGO staff members, and they were also the  only participants to mention the scientific basis of the Declaration. One explained:

*Before the formula was given to this HIV-positive mothers, nê, and then the problem it was they don’t know how to measure the formula and the babies got sick with vomiting and diarrhea. The Government researched more about formula, that the mothers should not give the formula to their babies. They must at least breastfeed, because of when they breastfeed, the breastmilk it’s available always, all the time, they don’t have to measure*. *.*. *sometimes the place where they stay, there’s no electricity there’s no running water. That is why it’s safer for them to breastfeed … That is why they’ve changed the guidelines of the mothers*. *.*. *They must breastfeed, all of them.* (NGO1, CHC2).

A strategy of re-emphasising old messages was how many HWs handled the new EBF-only imperative. Women explained that they had previously counseled HIV-infected women to either EBF for six months or EFF to prevent HIV transmission. NGO workers noted that now the emphasis was placed on providing all women, regardless of HIV status, the same advice:

*The government is saying when you are HIV positive or not, you have to breastfeed exclusively for the first six months*. *.*. *And we have to explain about the HIV, because there are lots of studies, researches about HIV. And it’s when they said, no, breastfeed exclusively. Something new is coming up every day. So whatever they are telling us to preach, we have to preach it the right way.* (NGO2, CHC2).

Nurses and WBOTs had also received training on infant feeding, although mostly in the context of PMTCT, WBOTS during their orientation and nurses during the course of their service. Neither group received training specific to the Declaration. Accordingly, their counseling included reference to both formula and EBF, although now with greater emphasis on exclusive breastfeeding.

Motivated HWs developed their own systems to remain updated on policy and to share what they learned from training opportunities*:* “When we come back, we share the information so that we are spreading one voice in the community” (NGO2, CHC2). When the free formula program ended, many emphasized the benefits of breastfeeding in counseling, as recalled from previous trainings, observations at the clinics, and personal experiences:

*[Breastfeeding] saves the mother’s time. It saves the mother’s money, because most of our people are not working. And it gives baby a very good growth. Babies who are breastfeeding don’t come to the doctor regularly. They don’t get sick every time.* (Nurse, CHC2).

### Counseling exclusivity: Making it exceptional

Most HW training was not grounded in the evidence base that informed the Declaration, and they interpreted EBF only promotion for themselves, often drawing on their knowledge from PMTCT training. This led to them to continue to focus particularly on HIV positive mothers. One nurse explained:

*Each and every one who comes to the clinic to give birth we advise them*. *.*. *breast is best. But we focus mainly on the positive mothers. We do talk to the negative mothers but…we focus on the positive mostly.* (Nurse, CHC4).

An unexpected finding was the difficulty HWs had in describing how to advise HIV negative women about exclusive breastfeeding. Rather than mentioning the nutritional benefits of exclusive breastfeeding, they focused on using condoms (“condomizing”) to avoid HIV seroconversion. A WBOT explained:

*You are breastfeeding and you’ve got your results as a negative mother and you also gave birth to a healthy baby child. So you start playing with your man. So we say condomize, because what will happen if you get infected and then that’s why it increase that the child can – this is the other way of the child getting HIV. So if you don’t condomize, you have the chance to be infected during the time of breastfeeding.* (WBOT3, CHC4).

When the PI asked one HW if she discouraged mixed feeding among HIV negative mothers, she struggled, understanding exclusive feeding's main objective to be HIV prevention. While she advocated breastfeeding, she could not see the point of emphasizing exclusive feeding for HIV negative women and their infants.

### Counseling dissonance: Promoting difficult behaviors

How does one promote a behavior one has not managed to achieve? Most HWs described their role, as counselors, to support mothers to make the final choice. “We can’t choose for them, some want to feed, some they don’t.” (WBOT, CHC4) They expressed a desire to give “correct” advice to protect both the infant and the mother and described breastfeeding as “best,” even though few had fed their own children this way. Most found it challenging to persuade women to breastfeed in ways that were consistent with the Declaration and had experienced the same barriers their patients described in terms of family pressures, work or school responsibilities, and mixed feeding norms. One nurse started mixed feeding her son at five months due to pressure from her husband:

*My husband was like, “No, when the child is crying*. *.*. *It’s because you don’t want to give baby food. He is hungry. Hey, you are all with this stuff for the clinic. Every time clinic says this, clinic says that. No man, you are going with these things of the clinic*. *.*. *Give the baby food.” He used to buy many boxes of Purity, Matabela [Local baby foods].* (Nurse, CHC2).

Rather than refer to the Declaration as an aid to their counseling, HWs described supporting each other, particular through teamwork, to address counseling challenges:

*They [mothers] encounter a lot of challenges; the [older nurses’] attitude is not good, but with the help of Mentor Mothers and the young staff at the ANC they are helping a lot, because we work hand in hand with them.* (Nurse, CHC2).

Health workers gave priority in counseling to helping mothers to support their infants generally, rather than to adhere to the Declaration’s EBF message. In some instances, they felt the EBF-only focus restricted open communication about choice. One counselor felt that the formula program discontinuation had removed a feeding option: “It seems like [the women] don’t have the choice. .. we’re speaking of options and choices, but this seems that they don’t have a choice”. (NGO2, CHC2) Other HWs discussed how the shift in clinic culture to promote EBF had resulted in some women falsely claiming that they were exclusively breastfeeding:

*If they find one person shouted at her, she won’t tell the truth*. *.*. *She’s so scared, so she thinks that everyone will shout at her, especially the (nursing) sisters. Sisters like to shout at people.* (CHC2).

### Changes in practices - breastfeeding ‘just like that’

Participants agreed that breastfeeding initiation had increased since the Declaration, although they had not necessarily planned this. A labor ward nurse explained how mothers changed their decisions after learning that free formula was no longer available:

*That time [pre-Declaration] there were plenty of mothers who were formula feeding. Now there are very few*. *.*. *When we ask them, did you bring the milk or what are you going to give your child? “Formula, I’m going to formula feed.” “Did you bring the milk?” “No. Doesn’t*. *.*. *doesn’t the government issue the milk?” “No. Not anymore. From 2011 it does not issue the milk.” “Oh. Okay, then I’ll breastfeed.” Just like that.* (Nurse, CHC3).

This nurse emphasized that last minute changes happened often, even though the formula program had ended four years earlier. Women’s last-minute decision-making reflected their general lack of preparedness, associated with late presentation for antenatal care and booking for delivery, and the lack of antenatal education that might have addressed feeding options prior to delivery.

Whether higher rates of breastfeeding initiation meant more EBF was unclear. The general impression across interviews was that mixed feeding (with maize gruel, tea and formula) remained a community norm, and increased breastfeeding was often framed around finance:

*Breastfeeding is the most popular, because they feel very comfortable and besides baby formula is a bit expensive these days. Some have actually given up baby formula and gone back to breastfeeding*. *.*. *they may end up mix-feeding them and the child may have complications in their digestive system.* (WBOT, CHC4).

Some community based HWs believed that removing the free formula option meant that women were more likely to introduce mixed feeding once they returned home. They emphasized this risk particularly for mothers returning to work or school, but also for those seeking to accommodate caregiving of infants by multiple people (primarily, but not only, the infant’s grandmother). In addition, HWs described the difficulties experienced by HIV positive mothers who had not disclosed:

*HIV positive ones are having a problem. Because sometimes they say: “What if I go back to work and I find out that my child is being fed something else and then I have to breastfeed?” Others don’t even breastfeed at all, they just give formula because they are scared.* (NGO1, CHC1).

The HWs gave a clear impression that HIV positive mothers continued to fear infecting their infants, despite the government’s 2015 policy of providing both mothers and infants with antiretroviral therapy (ART). Multiple HWs felt that the 2015 universal ART policy had resulted in better child survival.

*So many infants died those days. But now it’s better because we are using new guidelines. Each and every new HIV positive mum gets FDC [Fixed Dose Combination] which is 100 % suppressing the virus. So many children now, born from these mothers who are getting FDC are HIV negative. Even if they breastfeed, because virus is seriously suppressed.* (Nurse, CHC2).

Nevertheless, HW's reported that some mothers still opted to formula feed rather than risk mixed feeding by family members.

Not all study participants saw the Declaration as creating hardship for HIV positive mothers. One NGO counselor argued that the Declaration made the lives of HIV positive mothers easier, “relieving” women of the need to collect formula from the clinic and the stigma associated with this. Having government formula was previously a clear indicator that someone was HIV positive, and most HWs mentioned persisting HIV stigma in the community.

## Discussion

Breastfeeding has become increasingly normative since the Declaration, potentially reducing social barriers to exclusive breastfeeding. Observations of feeding behaviors by the HW confirmed a positive shift in breastfeeding uptake, consistent with recent statistics [18, 34]. This contrasts with patterns a decade earlier, when it was difficult to find exclusive breastfeeding in urban South Africa [35]. Even so, mixed feeding remains a strong community norm. This study also demonstrates that many mothers continue to opt for formula and seek HW advice on this, consistent with other studies conducted since the Declaration has been in effect [9, 34]. Early unplanned pregnancy, cooperative caregiving, four month unpaid maternity leave, food insecurity, and HIV remain barriers to EBF in South Africa [15, 19].

Even with limited knowledge of the Declaration’s evidence base, HWs had formed strong opinions about the policy’s contribution to increased breastfeeding. Facility based HWs perceived improvements in child survival, consistent with another recent study in Soweto [34]. The HWs in our study partially attributed this to the government’s provision of ART to all mothers. Our inclusion of other types of HWs introduced different perspectives. The WBOTs worried that formula removal was increasing mixed feeding among HIV positive mothers, a fear expressed by researchers when the Declaration was passed [26]. These contradictory opinions suggest that the context of care provision (facility versus community) may influence HW policy perspectives.

Health workers play a critical role in framing feeding choices. Behavioral theories, such as the Theory of Planned Behavior, can harness knowledge about the nutritional and developmental benefits of EBF to improve mothers’ attitudes, intentions and EBF behaviors [36]. Although HWs did not frame feeding choices on the basis of new knowledge resulting from the Declaration, their EBF and formula feeding knowledge seemed relatively high compared with government assessments of nutritional counseling by nurses. A government’s assessment of nurses’ knowledge of counseling messages to mothers whose children were not growing well found that less than 30% of nurses mentioned EBF for six months in two of four provinces [37]. Their greatest error seemed to be in diminishing the importance of EBF for infants not exposed to HIV, as identified in the other Soweto study [34]. Our study sample likely represented a more knowledgeable cohort of HWs than one might expect nationally, painting a more optimistic picture than elsewhere.

Health workers emphasis on breastmilk as a potential vector of HIV, as opposed to a source of nutrition, reflects South Africa’s long history with HIV. The likelihood of transmission is low with ART [4]. Johannesburg, the municipality where this study was conducted, has among the highest rates of ART initiation (92.1%) and Polymerase Chain Reaction (PCR) birth testing (70.3%) in the country [38]. The risk of seroconversion in the postnatal period exists, but HWs were likely overemphasizing this risk, creating unwarranted concern among women. Educating HWs about HIV risk communication could help them better serve mothers in Soweto [39]. In doing this, the cautionary notes of other scholars of risk communication should be heeded: scientific evidence should not be distorted [40] and social risks should not be discounted [41] in order to persuade women to adopt a single ideological position adopted by a scientific community or nation.

The Declaration’s contribution to confusion about the counseling role needs to be addressed. HWs felt that their counseling role was undermined by the single message. The closest description of what was happening comes from a comparison of HWs from Burkina Faso, Cameroon and Cambodia [42]; the researchers showed that rather than applying the authentic meaning of counseling to “help a mother decide what to do,” HWs practiced more or less prescriptive counseling based on country PMTCT guidelines and health systems. A similar observation was made in another study of HWs in South Africa [23], leading to questions about how HWs are trained to counsel. The HWs interviewed in this study were uneasy about prescribing behaviour to mothers, although many admitted to “preaching.” HWs described the ethical dilemmas in counseling HIV positive mothers to breastfeed when they believed they would mix feed, echoing an earlier sociological study on this topic in South Africa [22]. While HIV stigma remains a barrier for disclosure [15], such dilemmas are likely to arise for HWs placed in counseling roles. The pressure some HWs felt to “preach” government messages is incongruent with the principles of quality counseling.

### Limitations

A key study limitation relates to transferability. We conducted a limited number of interviews from three different types of HWs in four clinics. The likelihood of selection bias was high in terms of infant feeding knowledge and interest, making our findings about low knowledge and need for training particularly interesting, as we would expect a random sample of HWs to highlight findings closer to those of the government [37]. Multiple interviews would have enabled deeper probing about the intersections between HWs’ personal experience, medical knowledge and lay principles, which other scholars have found to shape counseling [42]. This may have enriched our understanding of cadre-related differences in perspectives. Most of the interviews were carried out in English by an outsider to the community, which may have resulted in social desirability bias. Health workers may have wanted to portray themselves is a positive light, given their previous negative portrayals reported by one facility manager. The level of self-reflection and critique in the interviews suggests that this was not the case. Despite such limitations, HWs’ behavioral observations of breastfeeding increases and other challenges faced by mothers were consistent with other studies in the community on infant feeding [34].

## Conclusions

In the Declaration, messages promoting exclusive breastfeeding were to be supported by enabling health systems and communities [16], and multilevel approaches to support EBF have been strongly endorsed [1]. As supports are not yet fully in place, we conclude with a focus on what can be done with HWs to create a more enabling environment. All women need support and advice, including HIV negative mothers, those opting for formula, and those currently mixed feeding. Until interventions adequately address socioecological barriers to EBF [1], all HWs need training in counseling that is responsive to the contexts of mothers.

We specifically suggest the following:

-Engage the increasingly diverse range of HWs in the health system in EBF promotion and counseling, giving then standardized training. This recommendation is supported by Jama and colleagues [9]. We highlight in particular WBOTs who government has not formally engaged in counseling pregnant mothers about infant feeding choices.

-Increase HWs access to information on changes in guidelines, explanations behind these, and the implications of changes, in a clear format. Platforms that enable dialogue and questions, as already exist in South Africa, need to be leveraged. For instance, NurseConnect provides nurses with free information and learning modules on their mobile phones [43] and could be used to learn about this topic.

-Moving forward, we recommend that the National Department of Health create channels for frontline HWs to provide consistent input on guidelines and to share their experiences as part of a feedback loop. As highlighted in Griswold’s recent column on reframing the breastfeeding narrative [44], health professionals working directly with families in their own communities have unique insights into the contextual factors that affect local behavior, and can positively influence policymaking. They are best able to identify barriers to EBF that need the greatest attention.

-Finally, HWs need training and support on how to communicate with mothers in the most effective manner, particularly in terms of risk communication [39]. Translation of the Declaration into practice needs clarification. Are frontline HWs expected to act as counselors, helping women decide from a range of options within their contexts, or are they expected to deliver and reinforce evidence-based information on a single feeding option? If the latter, or either way, we suggest that there is an urgent need to improve the support that mothers receive from HWs, in order to make infant feeding decisions that support their infants’ survival and ability to thrive.

## Abbreviations

ANC: Antenatal Care
ART: Antiretroviral Therapy
ARV: Antiretroviral
CHC: Community Health Clinic
CHW: Community Health Workers
EBF: Exclusive Breastfeeding
EFF: Exclusive Formula Feeding
HIV: Human Immunodeficiency Virus
HIVSA: HIV South Africa
HW: Health Workers
MM: Mentor Mother
NDOH: National Department of Health
NGO: Non-governmental organization
PCR: Polymerase Chain Reaction
PI: Primary Investigator
PMTCT: Prevention of Mother to Child Transmission
RAs: Research Assistants
WBOTs: Ward-based Outreach Teams
WHO: World Health Organization

## References

[1] Rollins NC, Bhandari N, Hajeebhoy N, Horton S, Lutter CK, Martines JC (2016). Why invest, and what it will take to improve breastfeeding practices?. Lancet.

[2] World Health Organization. The optimal duration of exclusive breastfeeding. In: *Report of an expert consultation*. Geneva: World Health Organization, 2002 march 28–30, 2001. http://www.who.int/nutrition/publications/optimal_duration_of_exc_bfeeding_report_eng.pdf Accessed 24 April 2018.

[3] World Health Organization. Guidelines on HIV and infant feeding. In: *2010. Principles and recommendations for infant feeding in the context of HIV and a summary of evidence*. Geneva: WHO; 2010. http://www.who.int/maternal_child_adolescent/documents/9789241599535/en/ Accessed 24 April 2018. 24501786

[4] World Health Organization (2016). United Nations Children’s fund. Guideline: updates on HIV and infant feeding: the duration of breastfeeding, and support from health services to improve feeding practices among mothers living with HIV.

[5] Victora CG, Bahl R, Barros AJ, Franca GV, Horton S, Krasevec J (2016). Breastfeeding in the 21st century: epidemiology, mechanisms, and lifelong effect. Lancet.

[6] World Health Organization (2010). UNICEF, USAID, AED, UCDAVIS, IFPRI. Indicators for assessing infant and young child feeding practices part 2: measurement.

[7] Hector D, King L, Webb K, Heywood P (2005). Factors affecting breastfeeding practices. Applying a conceptual framework. NSW Public Health Bull.

[8] Sibeko L, Coutsoudis A, Sp N, Gray-Donald K (2009). Mothers' infant feeding experiences: constraints and supports for optimal feeding in an HIV-impacted urban community in South Africa. Public Health Nutr.

[9] Jama NA, Wilford A, Masango Z, Haskins L, Coutsoudis A, Spies L (2017). Enablers and barriers to success among mothers planning to exclusively breastfeed for six months: a qualitative prospective cohort study in KwaZulu-Natal, South Africa. Int Breastfeed J.

[10] Mphego Z, Madiba S, Ntuli B (2014). The influence of the family on adherence to exclusive breastfeeding: experiences of women living in extended family households in poorly resourced communities of Mpumalanga Province, South Africa: child nutrition and feeding practices. Afr J Phys Health Educ Recreat Dance.

[11] Bland RM, Rollins NC, Coovadia HM, Coutsoudis A, Newell ML (2007). Infant feeding counselling for HIV-infected and uninfected women: appropriateness of choice and practice. B World Health Organ.

[12] Horwood C, Butler L, Barker P, Phakathi S, Haskins L, Grant M (2017). A continuous quality improvement intervention to improve the effectiveness of community health workers providing care to mothers and children: a cluster randomised controlled trial in South Africa. Hum Resour Health.

[13] Daniels K, Nor B, Jackson D, Ekstrom E, Doherty T (2010). Supervision of community peer counsellors for infant feeding in South Africa: an exploratory qualitative study. Hum Resour Health.

[14] Doherty T, Sanders D, Jackson D, Swanevelder S, Lombard C, Zembe W (2012). Early cessation of breastfeeding amongst women in South Africa: an area needing urgent attention to improve child health. BMC Pediatr.

[15] Du Plessis L, Peer N, Honikman S, English R, Padarath A, King JF, Mackie E, Casciola J (2016). Breastfeeding in South Africa: Are we making progress?. *South African Health Review.* Durban: health systems trust.

[16] Department of Health (2011). The Tshwane declaration of support for breastfeeding in South Africa. South Afr J Clin Nutr.

[17] World Health Organization (2010). *Summary of evidence for the revised WHO principles and recommendations on HIV and infant feeding*.

[18] National Department of Health, Statistics South Africa, South African Medical Research Council, ICF. *South Africa Demographic and Health Survey 2016: Key Indicators.* Pretoria, South Africa and Rockville, Maryland, USA: NDoH, Stats SA, SAMRC, and ICF, 2017. https://www.statssa.gov.za/publications/Report%2003-00-09/Report%2003-00-092016.pdf Accessed 20 April 2018.

[19] Lazarus R, Struthers H, Violari A (2013). Promoting safe infant feeding practices–the importance of structural, social and contextual factors in southern Africa. J Int AIDS Soc.

[20] Baker U, Tomson G, Some M, Kouyate B, Williams JM (2012). How to know what you need to do': a cross-country comparison of maternal health guidelines in Burkina Faso, Ghana and Tanzania. Implement Sci.

[21] Doherty T, Sanders D, Goga A, Jackson D (2011). Implications of the new WHO guidelines on HIV and infant feeding for child survival in South Africa. B World Health Organ..

[22] Seidel G (2004). Decisions and advice about infant feeding: findings from sociological work in KwaZulu-Natal, South Africa. Afr J AIDS Res.

[23] Buskens I, Jaffe A (2008). Demotivating infant feeding counselling encounters in southern Africa: do counsellors need more or different training?. AIDS Care.

[24] de Paoli MM, Mkwanazi NB, Richter LM, Rollins N (2008). Early cessation of breastfeeding to prevent postnatal transmission of HIV: a recommendation in need of guidance. Acta Paediatr.

[25] Lazarus R, Struthers H, Violari A (2009). Hopes, fears, knowledge and misunderstandings: responses of HIV-positive mothers to early knowledge of the status of their baby. AIDS Care.

[26] Saloojee H, Gray G, JA MI (2011). HIV **and** Infant feeding – one step forward, two steps back. South Afr J HIV Med.

[27] Dodgson JE (2017). About research: qualitative methodologies. J Hum Lact.

[28] Department of Health. *The 2012 National Antenatal Sentinel HIV and Herpes Simplex Type-2 Prevalence Survey*. 2014. Pretoria: Department of Health. https://www.health-e.org.za/wp-content/uploads/2014/05/ASHIVHerp_Report2014_22May2014.pdf Accessed 20 April 2018.

[29] Kinkel H-F, Marcus T, Bam N, Hugo J, Memon S (2013). Community oriented primary care in Tshwane District, South Africa: assessing the first phase of implementation. Afr J Prim Health Care Fam Med.

[30] Naledi T, Barron P, Schneider H, Padarath A, English R (2011). Primary health care in SA since 1994 and implications of the new vision for PHC re-engineering. **South African health review**.

[31] m2m (2015). Latest news [internet]. Cape town: m2m.

[32] Braun V, Clarke V (2006). Using thematic analysis in psychology. Qual Res Psychol.

[33] Fereday J, Muir-Cochrane E (2006). Demonstrating rigor using thematic Analysis : a hybrid approach of inductive and deductive coding and theme development. Int J Qual Methods.

[34] Mnyani CN, Tait CL, Armstrong J, Blaauw D, Chersich MF, Buchmann EJ (2016). Infant feeding knowledge, perceptions and practices among women with and without HIV in Johannesburg, South Africa: a survey in healthcare facilities. Int Breastfeed J.

[35] Sibeko L, Dhansay MA, Charlton KE, Johns T, Gray-Donald K (2005). Beliefs, attitudes, and practices of breastfeeding mothers from a periurban community in South Africa. J Hum Lact.

[36] Guo JL, Wang TF, Liao JY, Huang CM (2016). Efficacy of the theory of planned behavior in predicting breastfeeding: meta-analysis and structural equation modeling. App Nurs Res.

[37] The Presidency (2014). Final Evaluation Report: Short Diagnostic/Implementation Evaluation of Nutrition Interventions for Children from Conception to Age 5.

[38] Massyn N, Peer N, English R, Padarath A, Barron P, Day C (2016). District health barometer 2015/16.

[39] Bennett P, Calman K, Curtis S, Fischbacher-Smith D (2010). Risk communication and public health.

[40] Wolf JB (2007). Is breast really best? Risk and total motherhood in the national breastfeeding awareness campaign. Journal Health Polit Policy Law.

[41] Knaak SJ (2010). Contextualising risk, constructing choice: breastfeeding and good mothering in risk society. Health Risk Soc.

[42] Desclaux A, Alfieri C (2009). Counseling and choosing between infant-feeding options: overall limits and local interpretations by health care providers and women living with HIV in resource-poor countries (Burkina Faso, Cambodia, Cameroon). Soc Sci Med.

[43] Health-e. *SMS service helps nurses help mothers*. Health-e News [Internet]. 2016. https://www.health-e.org.za/2016/09/10/nurses-use-sms-service-help-mothers/ Accessed 24 April 2018.

[44] Griswold MK (2016). "you are not alone": toward equity in breastfeeding and skilled lactation care. J Hum Lact.

